# Understanding the properties of intermittent catheters to inform future development

**DOI:** 10.1177/09544119231178468

**Published:** 2023-06-10

**Authors:** Jessica V. Moore, Jane Burns, Nicola McClelland, James Quinn, Colin P McCoy

**Affiliations:** School of Pharmacy, Queen’s University Belfast, Belfast, Northern Ireland, UK

**Keywords:** Intermittent catheters, in vitro characterisation, lubricity, friction, urethral micro-trauma, pathogen displacement, urinary tract infections, biocompatibility

## Abstract

Despite the extensive use of intermittent catheters (ICs) in healthcare, various issues persist for long-term IC users, such as pain, discomfort, infection, and tissue damage, including strictures, scarring and micro-abrasions. A lubricous IC surface is considered necessary to reduce patient pain and trauma, and therefore is a primary focus of IC development to improve patient comfort. While an important consideration, other factors should be routinely investigated to inform future IC development. An array of in vitro tests should be employed to assess IC’s lubricity, biocompatibility and the risk of urinary tract infection development associated with their use. Herein, we highlight the importance of current in vitro characterisation techniques, the demand for optimisation and an unmet need to develop a universal ‘toolkit’ to assess IC properties.

## Graphical abstract

**Graphical abstract:**
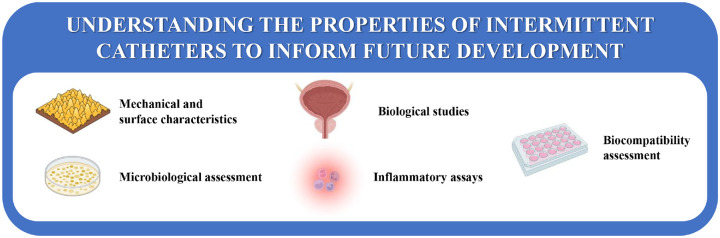

## Introduction

Urinary catheters are invaluable to patients suffering from urinary incontinence or urinary retention.^1^ They have been hugely advantageous to patients’ quality of life as they can go about their normal routines, providing freedom to work and socialise.^2^ Urinary catheters are the most commonly employed medical device in the UK.^3^ People of any age may require the use of a catheter due to underlying medical conditions, such as spinal cord injury (SCI), stroke and multiple sclerosis.^2,4,5^ There are two main types of urinary catheters: indwelling and intermittent. Indwelling catheters (IDCs) can remain in place for several days or weeks depending on the need and are held in position in the bladder by an inflated balloon, whereas intermittent catheters (ICs) are temporarily inserted into the bladder and removed once urine drainage is complete.

Typically, IDCs in situ for <30 days are considered short-term, and >30 days long-term or chronic use.^6^ Around 96 million IDCs are sold worldwide each year.^7^ The convenience associated with IDCs, avoiding repeated catheter insertion and not having to go to a bathroom, can be appealing.^8^ However, despite their medical necessity, one study found that of the 83 IDC users interviewed, 42% reported discomfort, 48% reported pain and 61% found restrictions in their daily life. Furthermore, 30% of the patients found catheterisation embarrassing.^9^ Chronic IDC users are anticipated to be incessantly bacteriuric,^10^ with resistant Gram-negative bacteria commonly found in the urine of long-term catheterised nursing home residents.^11,12^

Unlike indwelling catheterisation, intermittent catheterisation can either be performed by the patient themselves or a carer and occurs multiple times a day, usually four to six times.^13,14^ Intermittent catheterisation was reported to be the most common type of catheterisation used by patients with multiple sclerosis.^15^ Intermittent catheterisation is often preferred due to the reported increase in comfort and patient independence, with no need for drainage bags.^16,17^ Furthermore, it can improve patient quality of life with a decrease in the incidence of long-term urinary tract complications.^18,19^ Long-term intermittent catheterisation is associated with a decrease in complications such as prostatitis, the incidence of urinary stones and infection rate compared with long-term indwelling catheterisation.^14,20^ ICs are commonly made of polymers such as silicone, polyvinyl chloride (PVC), and latex. Silicone is considered to be one of the most biocompatible materials for catheter manufacturing. PVC is durable, flexible, low-cost and softens at body temperature to enhance comfort, however, skin sensitivity and allergies due to the leaching of plasticisers can occur.^21,22^

Nevertheless, repeated use of ICs has been associated with urethral trauma, false passages and strictures, especially in neurological patients, which not only affects their quality of life but can complicate subsequent treatments.^23–25^ Urethral strictures, which are narrowing of the urethra due to scar tissue, result in obstruction, weakening of the epithelium, urethritis and increased hospital visits.^26–28^ Discomfort is common, especially when catheterisation initially begins, and can worsen with patients’ anxiety surrounding the issue.^17^

**Figure 1.**
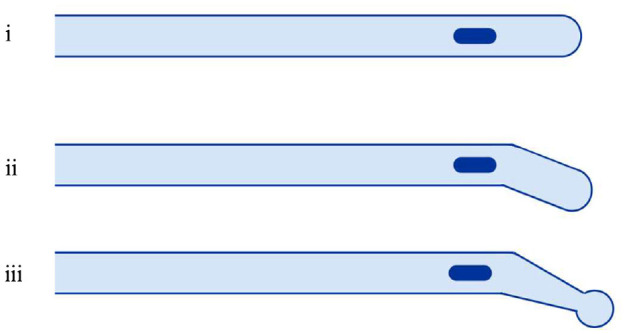
Schematic of catheters with (i) conventional tip, (ii) Coudé tip and, (iii) Tieman tip. Images drawn with bioRender 2022.

In response, choosing alternative catheter materials has been encouraged, such as silicone, which is associated with a lower risk of urethritis and urethral strictures.^29^ Catheter tips may also vary in shape; Coudé and Tieman angulated tips are deemed suitable for men with urethral strictures (Figure 1).^29^ Urethral strictures, bleeding and false passages are related to urethral insertion, therefore careful consideration is required to ensure selection of the most suitable catheter, along with the use of appropriate lubrication and positioning to aid catheter insertion.^29^

The use of hydrophilic-coated ICs is another approach to combat these complications. Most ICs have hydrophilic coatings, aimed at reducing friction between the catheter surface and urethral mucosa. Hydrophilic coatings are polymer layers capable of absorbing and binding water, resulting in a slippery and lubricated surface.^21^ A study by Clark et al. projected hydrophilic coated ICs to be more cost-effective in practice by reducing the occurrence of urinary tract infections (UTIs) per patient by ∼16%.^30^

**Figure 2.**
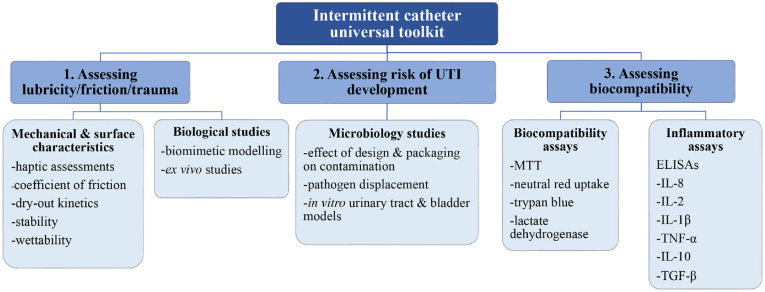
Suggested universal ‘toolkit’ to assess intermittent catheter properties.

Despite hydrophilic coatings enhancing the lubricity of the IC surface for improved insertion during intermittent catheterisation, issues with regard to coating stability and dry-out, bacteria transfer and cytotoxicity have been reported.^31^ Moreover, the risk of sensitisation to the coatings exists.^17^ Considering these issues, other elements should be routinely assessed to inform the future development of ICs. Numerous in vitro tests are widely published and are used to define surface properties and characteristics of both novel IDC and IC prototypes, however, there lacks consistency between investigations and relevance specifically to ICs. IC lubricity, biocompatibility and the risk of UTI development associated with their use should be investigated to enable the future evolution of more effective and patient-friendly ICs. Herein, we discuss current in vitro characterisation techniques, and highlight the importance of optimisation of current investigations and the need for a universal ‘toolkit’ to assess the properties of all ICs (Figure 2).

## Mechanical and surface characteristics

It is important to consider how an IC surface will interact with another surface in motion. Specifically, the interaction between the catheter surface and the urethra during catheterisation needs to be evaluated against the ease with which a catheter can be inserted and removed. This is a fundamental assessment indicating potential pain or discomfort a patient will experience during the catheterisation process. Many different testing methods have been employed to determine the frictional, mechanical and lubrication characteristics of IC surfaces. Assessment of coefficient of friction (CoF) is the standard tribology test; however, other well established surface characterisation approaches include haptic assessments, measurement of surface wettability, dry-out kinetics, catheter coating stability and in vitro models.^32,33^

### Why is lubricity relevant for ICs?

To conform with the American Society for Testing and Materials (ASTM) standards for urological application, ICs should possess certain physical and mechanical properties.^34^ A smooth, flexible and pliable surface is needed to manoeuvre through the urethral canal without perforating tissue, while retaining enough strength to withstand internal forces and prevent the catheter from kinking or collapsing during insertion.^34^ PVC, silicone, rubber, and novel PVC-free materials such as polyolefin-based elastomers are widely used in catheter manufacturing.^22^ Environmental issues regarding the overuse of plastic and safety concerns about phthalates found within many PVC products have led manufacturers to explore more sustainable options.^22,35,36^ IC surface properties can be enhanced by adding lubricous coatings. Polyvinyl pyrrolidone (PVP) is a common hydrophilic polymer applied to IC surfaces to reduce friction. The ability of PVP to bind water or saline allows it to act as a lubricant and through swelling of the polymer network it provides a lubricious surface for catheterisation.^37^ However, PVP is known to dry out rapidly and become adhesive, which can lead to difficult and painful catheterisation^38,39^ Therefore, the time for the patient to insert the IC is limited. Catheter users who have reduced range of motion and hand mobility due to illnesses such as multiple sclerosis, spinal injuries, and arthritis and elderly patients may find they have insufficient time to catheterise before the surface dries out.^40^

Lubricity plays an important role in the prevention of urethral trauma during catheterisation, as sub-optimum levels of surface lubricity will increase friction and wear.^41^ If a catheter’s surface is not well lubricated before use, frictional forces between the catheter’s surface and urethral mucosa may damage surrounding tissue. Long-term, repeated use of ICs is strongly associated with urethral trauma, which can result in inflammation, pain, and bleeding.^33^ Despite ICs being designed to minimise these undesirable effects, they are still limited by the surface’s durability to withstand friction forces, coating shearing that occurs during catheter insertion and withdrawal, and the ability to retain water at the surface for the duration of catheterisation.^31^

### In vitro testing methods

#### Haptic assessments

Haptic assessments are often used as a pre-clinical screening assessment during the initial development of catheter coating formulations and provide an indication of how catheter surfaces may perform in vivo. Haptic information allows assessors to identify preferences based on what they feel. This subjective feel of lubricity is important during patient catheterisation and may provide useful information as to why patients have preferences for one type of catheter over another. A catheter’s base material and surface coating will guide a person’s sensory evaluation, leading patients to choose a product that will provide them with a more comfortable user experience. This is a beneficial assessment that may prevent the researcher progressing to more regulated, time-consuming, and expensive clinical trials based on their findings.

Irwin et al. performed haptic assessments on three different formulations of coated PVC samples. Each formulation, intended for use as hydrophilic catheter coatings, was ranked on a scale of slippiness.^31^ Participants in the assessment were blinded to sample order and assignment, and each made three two-way comparisons between a test sample and a control. Samples were hydrated in deionised water for 30 s to mimic standard commercial hydration conditions and passed to the participant to rub the surface for 15 s. A Likert-type scale was used to rank each material in terms of slippiness: 1 a lot less slippery, 2 slightly less slippery, 3 slightly more slippery and 4 a lot more slippery. No significance in the subjective feeling of catheter lubricity between the control and test surfaces was observed highlighting a need for optimisation of human haptic assessments to enhance reliability. High variability in human tissue, the countersurface in this haptic assessment, may have an effect. Moreover, limitations of haptic assessments may include sample size, whether the assessors are catheter or non-catheter users, subjectivity and unvalidated Likert-type scales.^31^

#### Coefficient of friction

CoF testing evaluates the frictional force between two surfaces, generally using two different materials. A tribometer device is used to measure friction under controlled conditions.^42^ CoF is defined as the ratio between a frictional force and a normal force, using equation (1);

(1) $$\mathrm{CoF}=\frac{\mathrm{Frictional}\;\mathrm{Force}}{\mathrm{Normal}\;\mathrm{Force}}$$

The magnitude of the CoF is determined by factors such as the properties of the surface material, surface roughness and the presence of a lubricant.^43^ Materials with low CoF values are considered to be lubricous and therefore any friction between contact surfaces will be low.^44,45^ Two methods of friction coefficients can be measured: static and kinetic. Static coefficient is the ratio between the maximum frictional force prior to movement of the normal load, while kinetic coefficient measures the ratio of friction force during movement of the normal load. Catheter lubricity is measured using the kinetic method, where a catheter is fixed to a weight, accounting for the normal force, and then the weight is moved across the catheter’s surface.^46^ Furthermore, friction can be assessed under wet or dry conditions. Wet conditions are preferred and relevant for testing catheter lubricity as they are more representative of the in vivo urethral conditions.^32^ Hydrophilic coated catheters are known to exert lower CoF, compared to uncoated catheters, and have proven to reduce urethral trauma associated with friction.^31,34,47^ Kazmierska et al. tested seven commercially available catheters with different coating types against three counter faces. The lowest static and kinetic frictional forces were observed for the hydrogel-coated catheters.^32^ However, IC CoF evaluation is limited by many factors; it does not account for differences in different body types, for example, differences in urethral canal size, body temperature, moisture content, and the repeated frictional forces that occur during catheter insertion and withdrawal.

#### Dry-out kinetics

It is important to consider and examine whether a catheter’s surface will remain hydrated and lubricous for the length of time required to complete catheterisation. Studying dry-out kinetics has shown that when hydrated catheter surfaces are exposed to air, moisture is lost from the surface over time, and the longer the surface is exposed to the air, the more moisture it will lose.^31^ Additionally, hydrophilic catheter surfaces that have the capacity to retain water at their surfaces have proven to outperform uncoated, PVC catheters.^48^ Results also showed the higher the percentage of surface hydrophilicity, the more moisture was retained at the surface over the drying period, accounting for the higher affinity of water molecules bound to the catheter’s surface.^48^ Novel techniques other than surface coatings have been employed to try and overcome issues surrounding dry-out and coating-shedding. Other factors that may be attributed to catheter surface hydration levels include altering the chemical properties of the IC surface, the presence and thickness of surface coating and osmolality. In the UK, ConvaTec^®^ launched GentleCath™, a range of ICs with an exclusive surface called FeelClean Technology™, using amphiphilic surfactant technology, moisture is retained on the catheters surface without the need for a surface coating. Osmolality has been reported to influence catheter lubricity.^48,49^ Wellspect^®^ manufacture catheters that possess Urotonic™ Surface Technology, where the catheter surface is isotonic to urine and is anticipated to prevent hydrophilic coatings from being removed during catheterisation.^49,50^ This technology has been reported to prevent rapid dry-out and maintain a lubricous surface.

#### Catheter coating stability

Loss of catheter coating is a significant issue for manufacturers and patients and has been widely reported.^51^ Not only should IC coatings remain lubricious throughout catheterisation but should also uphold stability against the mechanical and shear forces of the urethral canal and bladder and withstand coating loss during catheter removal. Catheter coating loss at any point during catheterisation will decrease lubricity by exposing the uncoated surface beneath.^38^ Loss of coating can increase the risk of tissue irritation.^38,52^ The frequency of intermittent catheterisation, up to six times daily, could intensify these risks further.^53^

A range of catheter surfaces were evaluated by Pollard et al. for loss of coating using a texture analyser and agar model. A suite of commercial uncoated, hydrophilic coated and hydrophilic surface ICs were immersed in red dye to stain any potential coating on the surface. Each IC was inserted into 1% w/w agar for 2 min at 5 mm s^−1^, before removal from the agar at 5 mm s^−1^. Upon removal of the catheter, the agar was inspected for residual dye. Residual dye was present within the agar following removal of the coated ICs, indicating coating shedding.^54^ Application methods and surface modification techniques are major factors in determining the overall slippiness and adhesion properties of the coating.^52^ If a coating has poor abrasion resistance due to an inappropriate application technique, this could lead to coating loss, reverting surfaces back to their non-lubricous state.^52^ Developing novel hydrophilic surface technologies, such as the use of amphiphilic surfactants, may help provide lubricous IC surfaces without the need for an applied coating.^54^

#### Surface wettability

Wettability is a measure of surface energy, or under physiological conditions, how likely a surface will interact with surrounding fluids and tissue.^55^ Wettability properties of ICs are often quantified using tensiometry where a water droplet is placed on the surface; the angle of the water is then measured over timed intervals. A contact angle <90° is considered wettable whereas >90° is non-wettable. When a water droplet is placed on a surface, hydrogen bonding occurs between the water molecules and hydrophilic surface moieties, more hydrophilic surfaces will bind more water.^31^ Wettability is an important assessment for urinary catheters as materials that are non-wetting generally exhibit higher frictional forces between the catheter surface and interfacing tissue and fluids, increasing the risk of urethral trauma.^56,57^ Hydrophilic IC surfaces enhance surface wettability and are favourable for catheter coatings as they create a lubricous surface and decrease the risk of urethra trauma.^33^

#### Alternative model systems

Currently, the CoF standard frictional test of catheter surface lubricity measures the associated friction between a weight that is pulled over a catheter’s surface, however, it is unclear how relevant this is to the in vivo situation.^58^ Alternative biological model systems have been developed to mimic the urethral conditions, providing a more realistic model to test the lubricity, friction, and wear of catheter surfaces and coatings. An agar model was employed by Jones et al. to measure the surface lubricity of various ICs using tensile analysis, with the work required to insert and remove catheters from an agar model analysed. Results were compared with two IC surface characterisation techniques: dynamic contact angle as a measure of surface wettability and surface roughness, determined using atomic force microscopy.^59^ Results compared the relationship between ease of removal, receding contact angle and surface roughness, finding that the relationship between ease of removal and surface roughness was significant, but the relationship between ease of removal and receding contact angle was not significant.^59^ This study uniquely defined how catheter surface properties directly contribute to catheter lubricity, showing that as surface roughness decreased catheter lubricity increased.^59^

Additionally, Jones et al. used an agar and mucin-coated silicone tubing in vitro model to evaluate catheter lubricity during urethral insertion, simulating a method that is more clinically relevant.^60^ Catheter lubricity was quantified using a texture analyser, measuring the forces required for the insertion and removal of catheters from both in vitro models. The forces required for the insertion and removal of all catheters were greater in the simulated mucin model than in the agar substrate.^60^ Marmieri et al. used an agar filled test tube system to measure catheter lubricity. Here, a catheter was inserted into the agar and then removed with an attached weight.^61^ Results showed that hydrophilic coated surfaces were readily hydrated by the water within the agar compared to uncoated and plasma treated surfaces.^61^ The hydrophilic coated surface allowed the catheter to slide more freely through the agar due to the lubricating surface layer of water.^61^

## Biological studies

### Effect of long-term IC use on the uroepithelium

As discussed, CoF is often used to determine the lubricous properties of IC surfaces. The ISO CoF guideline (ISO 8295) is a standard test to determine the static and kinetic coefficient of friction between two surfaces, applicable to films and sheets, <0.5 mm thick and ‘non-sticky’ surfaces. However, this well-established method has limitations for testing of ICs due to the potential drying out of the IC coating resulting in ‘sticky’ surfaces and the absence of a physiological matrix. Catheter associated micro-trauma is extensively reported due to friction between the catheter surface and uroepithelium, therefore it is important to consider other frictional tests in addition to CoF investigations, as a reduction in CoF has been shown to reduce urethral micro-trauma.^33^

#### Biomimetic modelling

Humphreys et al. adapted the standard CoF method by including a human urethral epithelial cell-seeded model of the urethra as a countersurface for the catheter to examine in vitro urethral micro-trauma in relation to friction from intermittent catheterisation.^33^ 10 × 10 × 0.6 cm sheets made from Sylgard 184 PDMS were coated with 4 µg cm^−2^ fibronectin, to increase hydrophilicity, before seeding with 50 × 10^3^ human urethral cells cm^−2^ to represent the urethral mucosa. The cell-seeded sheets were placed into a bath of complete growth media at 37°C to replicate the moist environment of the urethra. 6 cm sections of ICs (uncoated, PVP coated and hydrophilic gel coated) were attached to a 200 g weight which applied 1.96 N force as the catheter moved at a rate of 200 mm min^−1^ horizontally across the cell-seeded sheets, mimicking catheterisation.^62^ A PDMS sheet without cells was placed before the cell-seeded PDMS sheet to demonstrate the difference in CoF measurements on the two different countersurfaces and the importance of considering urethral epithelium interaction with the IC surface. The cell-seeded sheets were then imaged after ‘catheterisation’ and the surface area of cells remaining attached to the PDMS surface on the catheterised section calculated. PVP-coated ICs were shown to exert mild epithelial damage whereas the uncoated and hydrophilic gel coated removed most of the cells from the PDMS surface. The hydrophilic gel coating was not chemically bound to the IC base material and was reported to form beads on the surface implying the importance of considering coating shedding as another factor in epithelial damage. Moreover, the PDMS dishes void of cells resulted in an overestimation of CoF values for the hydrophilic gel coated IC due to polymer-polymer interaction with the catheter base material surface at the rubber countersurface. As the hydrophilic gel coated catheters moved from the PDMS dishes without cells onto the cell seeded monolayer, a reduction in CoF was reported. This highlights that the standard CoF measurement between two surfaces has its limitations, and this adapted method may be more clinically relevant. Interestingly, images taken of cell damage after catheterisation were found not to correlate with the CoF measurements indicating the potential of various factors in urethral irritation and need for further development of the model.^33^

#### Ex vivo models

Kazmierska et al. acknowledged that despite the lubricity of urinary catheters being determined by frictional tests in line with ASTM standards, these tests are not relevant to the moist environment of the urethra.^32^ The presence of a liquid film has been shown to influence CoF values.^63^ Furthermore, the ASTM standards state that this frictional test method is not equivalent to ISO 8295–1995, and there should not be direct comparison between the two methods. The absence of standard specifications for investigating the surface lubricity of IC is evident.

A tribometer was modified to measure friction between polymer surfaces and tissue under wet conditions to mimic in vivo conditions. Kazmierska et al. tested uncoated and hydrogel coated catheters against polymethacrylate, porcine aorta and porcine urethra countersurfaces. Catheters were moved across the countersurfaces in a parallel plane at 1 cm s^−1^ in a vessel of distilled water at room temperature. Frictional forces were measured highest against the polymethacrylate and lowest against the porcine urethra countersurfaces. Furthermore, a difference in frictional results for each catheter was observed between the porcine aorta and porcine urethra tissue, indicating the importance of considering an appropriate countersurface relevant to the clinical application. The authors found lubricity of catheter surfaces measured in this modified tribometer set up was convenient, reproducible, objective and appropriately mimicked the in vivo process of catheterisation with regards to wet conditions and tissue countersurface.^32^

Tentor et al. recently described a complex ex vivo porcine lower urinary tract model to analyse IC performance and compared the model with a porcine in vivo study.^64^ The authors commented on the importance of developing effective in vitro and ex vivo models to assess catheter performance throughout the whole catheterisation process, unlike previous models that have primarily focused on evaluating one section of the process. The valuable information gathered from preliminary studies can be very beneficial and can guide the more complex and expensive in vivo and clinical studies.^64^

A porcine model was considered the most relevant model to study intermittent catheterisation due to its similarities with human physiology and anatomy. The study aimed to analyse the handling of the catheter, urine flow rate and flow stop, residual volume at flow stop, and the occurrence of mucosal suction when using different commercial ICs. Flow rate refers to urine flow through the catheter during voiding and the results from the study indicate the ICs displayed significantly different flow rates, attributed to varying IC eyehole position/size and lumen diameter. Premature removal of the IC during voiding can lead to a volume of urine remaining in the bladder, which is referred to as residual volume, and is considered a risk factor for UTI development.^64,65^ When the flow of urine first stops the user may perceive the bladder as empty and remove the catheter, however, repositioning the catheter may allow further urine drainage. Micro-trauma of the bladder mucosa can occur as a result of mucosal suction; the mucosa is abruptly sucked into the catheter through the eyehole causing tissue damage. Results showed mucosal suction occurred for all ICs tested. The ex vivo findings were supported by subsequent in vivo experiments carried out in pigs. Overall, this sophisticated model demonstrates how advanced ex vivo models can help bridge the gap between the more simplistic in vitro assays and expensive in vivo studies to evaluate IC performance.^64^

## Microbiological studies

### Are bacterial infections an issue and relevant toIC development?

For those requiring urinary catheterisation, single-use ICs are typically considered the gold standard for urine drainage due to less complications associated with intermittent catheterisation compared to IDC use; unlike IDCs, the residence time of ICs is too short to allow bacterial biofilm formation on their surfaces.^66–68^ IDCs are associated with frequent UTIs, blockages and more serious complications such as renal insufficiency, bladder stones and kidney stones.^67,69,70^ Although the incidence of UTIs in IC users is less than those who rely on IDCs, recurrent UTIs are still a significant complication associated with intermittent catheterisation for patients with SCI. A recent observational study found that these infections remain one of the top reasons SCI patients stop intermittent catheterisation.^69,71^

IC-associated UTIs are believed to be a result of the IC introducing bacteria into the urinary tract and bladder on insertion. Essentially, insertion of the IC can push or displace bacteria from the opening of the urethra (urethral meatus), which is typically colonised with a high concentration of bacteria, along the urethra into the bladder.^66,68,72^ Alternatively, exogenous bacteria from the environment can contaminate the catheter surface during preparation and insertion, further introducing bacteria into the sterile urinary tract.^73^ A study by Barford et al., although focused on IDCs, highlighted contamination of the catheter tip upon insertion is a major route for introducing bacteria into the bladder.^74^ Furthermore, a controlled clinical study by Kaye et al. revealed an approximate three-fold increase in the number of female patients experiencing bacteriuria following a single bladder catheterisation at childbirth compared to those who were not catheterised.^75^ Further suggesting that catheter-associated urinary tract infections (CAUTIs) can develop following a single catheterisation event, reiterating how ICs can also be associated with infection and the importance of assessing catheters’ pathogen displacement properties.^66,75^ Additionally, residual urine in the bladder due to incomplete voiding and IC-induced micro-trauma to the bladder and urethra are associated with UTI development.^64,65^ Increased volumes of residual urine left in the bladder following IC-aided voiding is considered a risk factor for UTIs, with the residual urine providing a haven for bacterial proliferation. This can occur due to incorrect user handling (i.e. the IC is removed before the bladder is empty), IC design and bladder shape.^64,65,76^

In the UK ICs are marketed almost entirely as single-use products, however, the reuse of ICs is considered normal in developing and some developed countries.^77^ A US study by Bolinger and Engberg found 56% of participants reused ICs a median of 20 times, which was attributed to the limited reimbursement users receive from medical insurers.^78^ Inadequate cleaning of ICs between use can increase the risk of complications, with the incidence of urethral trauma and UTIs reported to be between 70% and 80%, compared to 40%–60% for single-use catheters.^79^ More evidence-based research is required to determine if cleaning methods are suitable.^80^

Intermittent catheterisation can be sterile or clean. Typically, a sterile insertion technique is used by healthcare workers in an acute care hospital setting (e.g. wearing sterile gloves and periurethral cleaning before insertion), while clean self-intermittent catheterisation is considered acceptable and more convenient in a non-acute setting.^81,82^ Clean intermittent catheterisation simply involves washing the hands and genitalia before catheter insertion, however, some believe this may not be sufficient to prevent bacterial contamination.^68,73^ In response, a selection of ICs have been developed to facilitate a ‘no-touch’ insertion method to make self-catheterisation a more aseptic process. These ICs can employ closed, no-touch systems with sleeves that cover and protect the entire length of the IC to prevent the user from touching the sterile surface on preparation and insertion.^73,83^ Additionally, the inclusion of an insertion or introducer tip to the IC design can further aid aseptic catheterisation with the introducer tip first being inserted approximately 1.5 cm into the opening of the urethra, preventing the IC tip from coming in contact with the urethral meatus which is typically colonised with a high concentration of bacteria.^72,83^

### In vitro studies to assess the risk of IC-associated urinary tract infections

There are a wide range of in vitro tests and models that have been developed to assess IDCs’ ability to prevent CAUTIs by investigating their resistance to bacterial colonisation, biofilm formation and encrustation. In a recent review, Cortese et al. commented on the lack of in vitro tests or models for ICs and the need for the development of standardised tests to assess individual ICs, helping to drive innovation to ultimately improve user experience.^72^

#### Effect of IC design and packaging on bacterial contamination

In vitro tests have been developed to assess if different IC designs and packaging influence the clean intermittent catheterisation process. An in vitro model was designed by Hudson and Murahata to assess if the ‘no-touch’ insertion technique influences the level of contamination transmitted to ICs.^73^ Briefly, preparation for insertion of a selection of ICs were mimicked using gloved hands contaminated with a defined *Staphylococcus aureus* or *Escherichia coli* inoculum (1 × 10^5^–1 × 10^6^ cfu mL^−1^), and the number of viable bacteria transferred to the ICs was quantified. Five catheters were assessed: three commercial hydrophilic ICs, one handled partially with the packaging and two ‘no-touch’ ICs which have a sleeve that covers the length of the catheter and self-contained gel lubricant. The study revealed a significant reduction in the number of viable bacteria recovered from the ICs using the ‘no-touch’ system (∼5 cfu) compared to the standard hydrophilic ICs (≤440 cfu), suggesting the use of a ‘no-touch’ IC insertion technique can make intermittent self-catheterisation a more aseptic process. Thus, potentially reducing exogenous bacteria entering the urinary tract and the incidence of UTIs.^73^

Holland and Fish employed the similar ‘contaminated hands’ in vitro assay described by Hudson and Murahata, using *Pseudomonas aeruginosa* as the challenge microorganism to assess the effectiveness of their ‘touchless’ IC, which includes a no-touch sleeve and an introducer tip. They also designed an effective distal urethral/meatus model, or ‘insertion tip test’, simply comprising a Petri dish containing *P. aeruginosa-*inoculated agar, with bore holes through both the agar and Petri dish. The IC tip was then pushed through the inoculated agar and out through the opening in the dish, representing the IC being inserted through a contaminated urethral meatus into the urethra. The tip of the IC pushed through (3 cm) was cut and the bacteria on the catheter surface recovered and quantified. The presence of the introducer tip reduced the number of viable *P. aeruginosa* contaminating the catheter tip, with approximately 12-fold (22–1.8 cfu) less bacteria on the touchless IC compared to a standard IC with no protective sleeve or introducer tip.^83^

These simple in vitro assays could be readily used to assess if the IC’s design and packaging influences the catheter’s sterility during the preparation and insertion process, with the degree of bacterial contamination quantified.

#### Pathogen displacement

**Figure 3.**
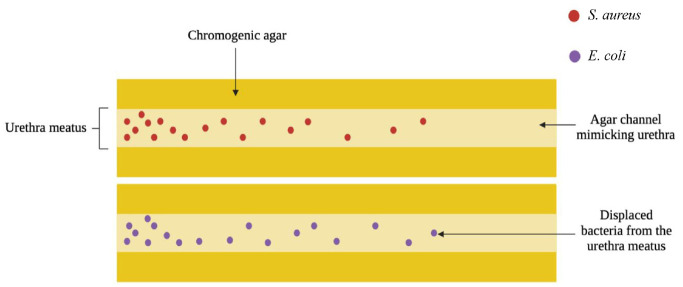
Diagram depicting novel in vitro urethra model showing how pathogens can be displaced during intermittent catheterisation. Adapted from Cortese et al.^66^ Images drawn with bioRender 2022.

Cortese et al. developed an in vitro urethral model to mimic the displacement of bacteria from the urethral meatus along the urethra upon insertion of an IC.^66^ The authors aimed to develop a reproducible urethral model that could be readily assembled in any laboratory, without the need for specialist equipment or uncommon bacteria strains. The uropathogens *S. aureus* and *E. coli* were chosen. Briefly, the model consists of chromogenic agar with preformed channels of sufficient diameter to allow insertion of an IC. The opening of the channel, representing the urethral meatus, is inoculated with bacteria to a depth of 1 cm and left for 30 min to allow absorption by the agar. The IC is then inserted into the channel and left in place for 30 s to mimic voiding of the bladder. The IC is removed and the agar left for 30 min at room temperature to allow absorption of any moisture. Following 24 h incubation, pink *S. aureus* colonies formed on the CHROMagar™ agar, which is selective for this bacterium and other staphylococcal species, and blue/purple colonies formed on the Harlequin™ agar, which is selective for *E. coli* and other faecal coliforms. This visual allows for immediate qualitative evaluation of bacteria displacement caused by IC insertion and removal (Figure 3). For quantification of viable bacteria, each channel is then dissected into octants, with the first octant being the opening of the channel. Each octant is sonicated in media, which is then diluted, and plated. The distribution of the colonies along the channels for the different catheters can be readily compared, which may be useful for informing the development of new ICs that displace bacteria to a lesser extent, potentially reducing UTI occurrence.^66^

### Adaption of current in vitro models specific to the study of indwelling catheter-associated urinary tract infections – why are they relevant?

There are several in vitro tests and anatomical models that have been used to assess IDCs’ ability to resist microbial colonisation and their susceptibility to blockage. While the focus of this review is on ICs, there is currently a lack of in vitro tests that can be used to assess IC performance. Therefore, IDC models have been included for context, with the potential for these models to be adapted to study the intermittent catheterisation process.

#### Urinary tract and bladder models

Gaonkar et al. developed an in vitro urinary tract model to investigate the migration of bacteria along the outer surfaces of IDCs impregnated with antiseptics.^84^ The model comprised two tubes, with the narrower upper tube leading into the lower tube which was used to collect the urine. A 6 cm catheter segment sealed at either end, to prevent intraluminal migration of the bacteria, was placed inside the upper tube that had been plugged at the bottom. Agar was poured into the space between the catheter and tube walls, and when solidified, the plug was removed exposing the catheter. A 1 cm section at the top of the catheter was also left uncovered, representing the bladder, and the bottom of the agar surrounding the exposed catheter represented the meatus. The meatus was inoculated with bacteria (10^7^ cfu mL^−1^) daily and fresh urine was added to the bladder. The meatus and bladder were cultured daily and at the point microbial growth was detected in the bladder, the catheter was removed and the presence of bacteria on the catheter surface confirmed. The time taken for the bacteria to reach the bladder was recorded, with the antiseptic-impregnated catheters displaying an extended time compared to the control of up to 25 days.^84^

Williams and Stickler and Morgan  adapted the model first described by Gaonkar et al. , suggesting the migration of bacteria along the original urethral model was accelerated due to physical forces at the agar-glass interface and the integrity of the agar column component may have been compromised over the extended test periods (up to 25 days).^85^ Thus, the modified model omitted urine, used chromogenic agar and was stopped after day 7. Use of the model to assess the migration of different bacteria over the surface of a silicone catheter revealed swarming species reached the bladder faster than swimming species, while non-motile species did not reach the bladder within the 7-day test period. Furthermore, both triclosan- and nitrofurazone-impregnated catheters were shown to inhibit the migration of swarming *Proteus mirabilis* from the meatus to the bladder by 7 days.^85^

The in vitro bladder model first described by Stickler et al. in 1999 is a well-established infection model still used today to study bacterial colonisation, encrustation and subsequent blockage of IDCs under flow conditions, allowing the effect of various antimicrobial strategies on the development of CAUTIs to be investigated.^86^ The model is composed of an open-ended sterile glass ‘bladder’, with a catheter, typically an indwelling Foley catheter, inserted through the bottom opening and the catheter balloon inflated to hold it in place. A defined bacterial inoculum is added to the bladder at the start of the experiment and sterile artificial urine is supplied to the bladder at a defined rate controlled by a peristaltic pump to mimic urine movement from the ureters into the bladder. When the level of urine in the bladder reaches the eyelet of the catheter, it drains into a sterile collection bag. The model can continue for days until the catheter eyehole blocks due to the formation of a crystalline biofilm.^86^

Barford et al. adapted the conventional in vitro bladder model described to include a lower urinary tract section to allow investigation of the potential ways bacteria can gain entry to the bladder during catheterisation. Attached to the bottom opening of the bladder was a glass tube representing the urethra, with a latex cuff at the distal end that was inoculated with varying concentrations of *E. coli* (3 × 10^4^, 3 × 10^3^ and 3 × 10^2^ cfu mL^−1^) to represent the meatus. Unlike the original Stickler bladder model, the bladder was not inoculated at the start of the experiment. Instead, the meatus section was inoculated, therefore bacteria detected in the bladder contents originated from here. Sterile catheters were then inserted into the bladder, passing through the inoculated meatus along the urethral channel into the bladder, where it was held in place by a rubber cuff. The flow of sterile urine into the bladder was started and after 24 h the experiment was stopped. Urine samples from the bladder and catheter bags were taken periodically (2, 4, 6, 8, 10, 24 h) and cultured. All samples showed the presence of *E. coli* in the bladder following catheter insertion, with all samples in the bladder colonised with a high concentration of bacteria (∼10^8^ cfu mL^−1^) by 24 h. A separate experiment which inoculated the meatus following insertion showed no bacteria on the catheter surface, suggesting ascension of the bacteria along the surface to the bladder did not occur, confirming in this model, bacterial growth in the bladder was due to displacement of bacteria from the meatus upon catheter insertion.^74^

The in vitro model developed by Barford et al. would be the model most amenable to adaptation to allow investigation of the effect of repeated intermittent catheterisation on UTI development. Unlike other in vitro bladder models, this model has a glass urethra tube attached to the bottom opening of the bladder, with the entrance of this tube inoculated to mimic a contaminated meatus. Single-use ICs could be inserted up to six times a day into the model, passing through the contaminated meatus, along the urethra and into the bladder for drainage to occur. Any displaced bacteria contaminating the IC tip will be introduced into the bladder, with the potential for some bacteria remaining, following ‘voiding’, contaminating residual urine remaining in the bladder; the presence of residual urine in the bladder is a known risk factor for UTI development.^64,65,76^ Following repeated intermittent catheterisation and potential contamination, a significant bacterial inoculum may establish in the bladder. The bladder contents could be regularly sampled to determine if/when the model becomes infected. Adaptation of well-accepted IDC models and the development of IC specific models should be considered in response to the dearth of in vitro tests available to assess IC-associated UTIs.

## Biocompatibility

### Why is cell compatibility relevant for ICs?

ICs are typically used several times a day, meaning that whilst each catheter does not remain in situ for long, the patient’s urethral tract and bladder still experience prolonged, cumulative exposure to ICs. It is therefore essential that the catheter itself, as well as any coatings or lubricants used to facilitate IC insertion, are biologically compatible with the uroepithelial such that they can remain in situ without causing tissue damage or inflammation.

The majority of ICs are comprised of PVC, latex or silicone elastomer.^87^ From a biocompatibility perspective, both PVC and silicone elastomer are regarded as good candidates for ICs due to their low cytotoxicity.^52,88–90^ On the other hand latex, as well as being a common cause of irritant contact dermatitis, has been shown to impair cell growth in cell cultures exposed to latex catheter eluate.^91,92^

Recent years have seen significant innovation in catheter coatings, in a move to not only improve lubricity but to also combat the incidence of UTI.^93^ In order to improve patient outcomes, it is important to ensure that any materials added to ICs to prevent infection do not pose a risk to patient health. The biological evaluation of ICs is governed by ISO guidelines, which consist of 20 different sections that each address separate aspects of medical devices. From a cell compatibility perspective, ISO 10993-5:2009 is a well-established guideline representing an excellent preliminary indicator of whether an IC will pose a risk to human health, making it the gold standard for in vitro cytotoxicity testing.^94^ The standard demands that ICs are subject to rigorous cytotoxicity testing to ensure that any materials that the human body comes into contact with, do not elicit a cytotoxic response. Some novel IDC antimicrobial coatings include the use of silver nanoparticles (NPs) as a broad spectrum antimicrobial, this however warrants caution as silver NPs are known to exhibit dose-dependent cytotoxicity in vitro.^95–97^

#### Methods to assess cell compatibility

The most commonly utilised test within this standard is the 3-(4,5-dimethylthiazol-2-yl)−2,5-diphenyltetrazoliumbromide (MTT) cytotoxicity test. This involves exposing a monolayer of cells to the materials in question for a defined period. MTT assesses cell viability by measuring the metabolism of viable cells which convert the yellow water-soluble MTT into purple formazan crystals. The number of viable cells directly correlates with the intensity of the purple formazan, which can be measured photometrically.^94^ A reduction in cell viability should be considered an early warning sign of potential cytotoxicity and inform further development of the IC. Samples can be assessed either in direct contact with cells or by adding a known quantity of sample extract into the cell media, which would be applicable for IC coatings or lubricants. The standard also incorporates a grading system, in which cell lines are microscopically examined for changes in cell morphology, cell lysis and/or membrane integrity, thereby providing a qualitative perspective to accompany the photometric determination.^52^

In spite of its advantages and relevance in the development of ICs, the MTT assay renders cell lines used non-viable and so isn’t optional if real-time assessment is required, such as mirroring repetitive intermittent catheterisation over time.^98^ Other tetrazolium-based approaches that assess mitochondrial activity include the XTT and WST-1 assays, which operate under similar principles as the MTT assay, without the necessity to solubilise cells prior to photometric detection.^99,100^ Alternative non-tetrazolium-based photometric assays which evaluate cellular metabolism include the resazurin assay, which permits cell lines to be repeatedly tested unlike the MTT assay, making it more appropriate for longer-term IC studies.^100,101^ The neutral red uptake assay is another well-established means of assessing cell viability, although it differs in the mechanism by which it detects cytotoxicity. In viable cells, neutral red can be incorporated into cell lysosomes, but the ability of cells to do so diminishes as cells are exposed to a cytotoxic onslaught, ultimately leading to loss of lysosomal integrity and cell death. A washing step is then performed, removing any neutral red that hasn’t entered cell lysosomes. The amount of neutral red can subsequently be quantified and correlated with cell viability.^102^

Trypan blue is another dye typically used to evaluate cell integrity, the principle here being that viable cells are able to stop the uptake of trypan blue as they have intact cell membranes. Cell lines exposed to ICs with cytotoxic potential will lose membrane integrity as cells begin to die, allowing trypan blue to bypass the cell membrane. Cells can then be counted by hemocytometry and the proportion of non-viable cells, which appear blue from trypan blue uptake, can be determined.^103^ Despite its use as a straightforward means of evaluating cytotoxic potential, over time cells will eventually uptake trypan blue leading to overestimation in IC cytotoxicity.^104^

Lactate dehydrogenase (LDH) is a cellular enzyme expressed in most body tissues including the urinary tract. Another method to assess cytotoxicity is measuring loss of membrane integrity. When cells are rendered non-viable, LDH is released from the cells into the surrounding medium, which is then indirectly quantified. The quantity of LDH release is proportional to the damage to the plasma membrane, allowing the assay to serve as an indicator of cytotoxicity.^105^

The authors believe MTT should remain the gold standard for assessment of cytotoxic potential, as it allows for accurate comparison between different materials used in ICs.^106,107^ It is suggested that other biocompatibility assays should still be conducted such as the resazurin assay, not only to support results achieved from MTT assays but to ensure that a variety of indicators of cytotoxicity are appraised. Whilst in vitro evaluation of IC is an important indicator in ascertaining whether the material poses a cytotoxic risk, it is not conclusive, and represents only a portion of the myriad of testing conducted under ISO guidelines before an IC meets the evaluation endpoint for urinary catheters.^108^

### Inflammation

#### Why is inflammation relevant for ICs?

No material can be considered truly biocompatible and so as soon as foreign materials such as ICs are exposed to the human body, an inflammatory response is initiated. Inflammation is a series of complex biological mechanisms that involves the recruitment of the host’s immune system to protect them from potential harm.^109^ This is normally transient, with excessive inflammation subsiding when the material is removed. However, even when using ICs, which are typically considered biocompatible, prolonged IC exposure through repetitive intermittent catheterisation can result in excessive inflammation causing adverse effects which compromise patient safety.^110^

Literature has shown that repeated IC insertion can induce urethritis, likely as a result of both blunt trauma upon insertion and removal, as well as from persistent inflammatory stimuli from IC usage.^18^ Another complication arising from IC use is urethral bleeding, which affects as many as one-third of patients and is typically seen at the onset of IC use.^23^ Chronic inflammation as a result of long-term IC use may result in the formation of urethral strictures, in which there is a narrowing of the urethral opening at the external meatus. If not addressed, the inflammation may spread to the surrounding spongiofibrosis tissue and the resulting deposition of scar tissue will lead to long-term narrowing of the urethral lumen.^17^ In an effort to ensure that the aforementioned adverse events do not occur, thorough testing of ICs must be undertaken.

#### Methods to assess inflammation

Due to the complexity of the inflammatory cascade, a number of targets for assessing the inflammatory response can be exploited to determine whether exposure to an IC is causing excessive inflammation. Enzyme-linked immunosorbent assays (ELISAs) are commonly used immunological assays which can quantify specific antibodies which are known to promote inflammation. IDCs with silver NP containing coatings are known to induce pro-inflammatory markers such as interleukin-8 and promote ROS production, with smaller NPs observed to have the highest pro-inflammatory effect.^111^ Pro-inflammatory molecules such as IL-2, IL-1β and TNF-α can be directly assessed using ELISAs and serve as a useful indicator of the inflammatory profile of in vitro cell lines exposed to ICs.^112^

Macrophages play an important role in the inflammatory response against ICs and upon stimulation, can be generally classified into two subpopulations, M1 and M2 macrophages.^113^ M1 macrophages are typically associated with an unfavourable inflammatory response, causing excessive inflammation and tissue damage, whereas M2 macrophages are considered anti-inflammatory, releasing an array of anti-inflammatory factors such as IL-10 and TGF-β.^114^ Researchers have utilised ELISA assays to evaluate macrophage activation, measuring the release of various pro-inflammatory and anti-inflammatory factors to ascertain what type of activation macrophages have undergone.^115^ Ideally, all ICs and surface additives should promote M2 macrophage activation to prevent tissue damage and reduce the likelihood of adverse events to patients. Assessment of inflammatory markers serves as an excellent insight into ascertaining if an IC in question has the potential to cause adverse effects as a result of excessive inflammation.

## Conclusions

A wide array of in vitro tests are available to investigate surface properties in the development of novel ICs. However, it is important to consider all relevant characteristics concerning lubricity, biocompatibility and the risk of UTI development associated with IC use. Inconsistency in testing parameters and standards within these assessments makes it difficult for direct comparisons between products and novel prototypes to be made. This review therefore details a universal ‘toolkit’ that will facilitate the routine assessment of IC properties, encouraging the consistent, standardised and relevant testing of ICs to inform future development. To adequately address each of the key issues associated with intermittent catheterisation, all three areas classified in the universal ‘toolkit’ should be considered. The tests chosen will likely be influenced by the resources and capabilities of each specific laboratory performing the studies.

When assessing IC lubricity or friction, the CoF assay is the standard lubricity test used to measure friction, however it fails to represent moist urethral conditions. The biomimetic model described by Humphreys et al.^33^ or the tribological studies detailed by Kazmierska et al.,^32^ provide alternative methods to assess lubricity and friction, with these methods better mimicking in vivo conditions. When assessing the risk of IC-associated UTI development, in vitro urinary tract and bladder models are sophisticated assays that have potential to allow the effect of repeated intermittent catheterisation on IC pathogen displacement and infection development to be investigated. However, these techniques require specialised equipment and are time consuming, which may make these models unfeasible. In contrast, the in vitro urethral model developed by Cortese et al.^66^ allows the effect of intermittent catheterisation on pathogen displacement to be assessed and can be readily assembled in any laboratory without the need for specialist equipment. As a measure of biocompatibility, the MTT assay can effectively evaluate IC surfaces that will be in direct contact with the uroepithelium for potential cytotoxicity. Overall, the universal ‘toolkit’ provides a selection of routine tests that allow the properties of ICs to be assessed, thus aiding the future development and evolution of more effective and patient-friendly ICs.
